# Recovery of Stabilizer Glass in Innovative MBT Installation—An Analasys of New Technological Procedure

**DOI:** 10.3390/ma13061356

**Published:** 2020-03-17

**Authors:** Jacek Połomka, Andrzej Jędrczak, Sylwia Myszograj

**Affiliations:** 1 Regional Municipal Waste Treatment Plant in Marszów, 68-200 Marszów, Poland; j.polomka@marszow.pl; 2 Institute of Environmental Engineering, University of Zielona Góra, Licealna 9, 65-417 Zielona Góra, Poland; A.Jedrczak@iis.uz.zgora.pl

**Keywords:** municipal waste, MBT installations, glass recovery

## Abstract

The data published by the European Container Glass Federation shows that the EU28 average collection rate for recycling of glass containers has grown to a rate of 76%. However the stabilizer produced at mechanical-biological treatment (MBT) installations at landfills still contains large amounts of recyclable glass. An industrial-scale study has been undertaken in order to assess the possibility of recovering this glass from the stabilizer. A new pilot installation was built at the MBT plant in Marszów, Poland. Tests were conducted on stabilizer samples produced at the plant (13 samples) and others collected from several MBT plants based in Poland (six samples). Processing the stabilizer on the designed line made it possible to recover on average 68.4 ± 7.0% of the glass contained in it from Marszów samples and 58.4 ± 14.2% in the case of samples acquired from other MBT installations. It is demonstrated that the concentrate quality largely depends on the stabilizer’s moisture content. A concentrate with glass content from 98.0% to 99.5% was obtained for samples of low-moisture stabilizers (for 14 out of 19 samples). The product was accepted by glass recycling plants due to its low level of contamination with other materials and its appropriate particle size.

## 1. Introduction

Glass is a fully recyclable material. Used glass containers can be recycled many times over without compromising their quality. At the same time, this enables one to save energy and protect the environment. Their use represents a 3% reduction in the fossil energy consumption used in glass manufacturing for every 10% of cullet as a result of replacement of natural raw materials, reduction in the amount of municipal waste, water consumption, extraction of raw materials and reduction in the production of greenhouse gases [1]. 

**Figure 1:**
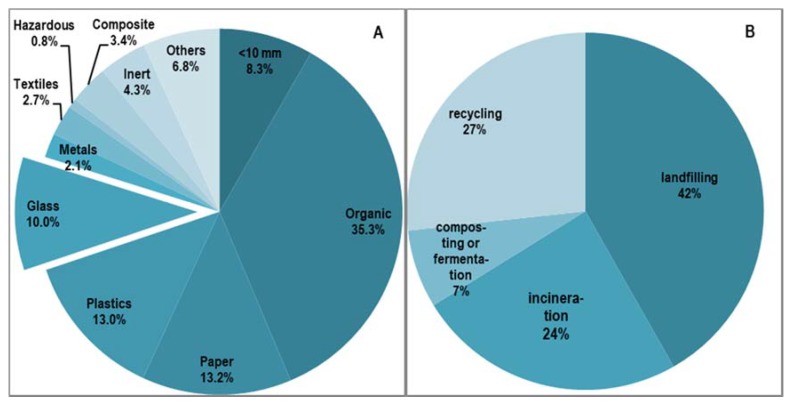
Morphological composition (National Waste Management Plan 2022) (**A**) [3] and ways of managing the MSW generated in Poland in 2017 (Directive 2008/98/EC) (**B**) [4].

In Poland, some 11.97 million tonnes of the municipal solid waste (MSW) was generated in 2017 [2]. The organic waste share amounted on average to 35.3%. The share of packaging waste (from paper, metal, glass, plastics) constituted 38.3% in 2017, including 10.0% of packaging glass (Figure 1A) (National Waste Management Plan 2022) [3]. About 61.3% of the MSW produced this year was sent to MBT plants for valorization. 

To organic or material recycling was subjected 33.8% of the waste. The share of landfilled waste stood at 41.8% and that sent to incineration plants or cement plants amounted to 24.4% (Figure 1B). Legal obligations for the management of municipal waste (household and similar waste) are defined in the Waste Framework Directive. One of the obligations arising from this document is to achieve the target of transfer of 50% of municipal waste for reuse or recycling by 2020 in all EU countries (Article 11(2) letter a) (Directive 2008/98/EC) [4]. The provisions of this Article were clarified in the Commission Decision of 18 November 2011, establishing rules and calculation methods for recycling and preparation for reuse rates of these municipal waste fractions (2011/753/UE) [4].

Through provisions in Poland’s new Act on Maintaining Cleanliness and Order in Municipalities (Article 3b (1)) (Journal of Laws 2012, item 391) [5] and in the Regulation on Recycling of Waste (Journal of Laws 2012, item 645) [6], the country chose the second variant of calculating the rate of recycling and preparation for reuse of paper, metals, plastics and glass. The level of recycling and preparation for reuse of these municipal waste fractions amounted to 28% in Poland in 2016 (Report NWMP, 2019) [7], reaching the value required for the year (18%) and for 2017 (20%), specified in the Regulation of the Minister of the Environment (Journal of Laws 2016, item 2167) [8]. 

In 2018, Poland was set to reach the 30% recycling level, and in 2019—40%. According to EC calculations, 14 EU member states, including Poland, will not reach the 50% threshold for recycling municipal waste (report COM 2018) [9]. Achieving the target in 2020 is therefore a major challenge that will drive the waste sector on the trajectory of developing technologies for recovering valuable packaging waste contained in the MSW. 

The recent amendment to the Waste Framework Directive introduced new, more ambitious targets for recycling municipal waste. By 2025, 55% of municipal waste should be recycled, 60% by 2030 and 65% by 2035 (Directive 2018/851) [10]. New recycling targets were also set for packaging waste. The adopted recycling targets for glass by 2025 (70%) and 2030 (75%) will be difficult to meet (Directive 2018/852) [11]. This means that a lot still remains to be done. 

Bearing in mind the need to achieve the required levels of waste recycling, the Ministry of Environment has already taken numerous actions, including legislative ones, to improve the quality of raw materials for recycling, such as the introduction of uniform standards for selective collection of municipal waste across the country (entered into force on 1 July 2017) (Journal of Law, 2017 item 19) [12], or increasing the rates of fees for landfilling waste that should be recycled (Journal of Law, 2017 item 2490) [13]. It will also be necessary to modify the technology in the existing MBT installations.

At the end of 2016, there were 192 MBT installations operating in Poland with a throughput capacity of approximately 11 million tonnes of waste per year. The dominant technology applied in these installations is the one in which in the mechanical part, an Organic Fraction of Municipal Solid Waste (OFMSW, fraction < 80 mm) is separated on a sieve from the mixed municipal solid waste (MSW) and biostabilized under aerobic conditions (composting). The end product (stabilizer) is disposed of in a landfill. The total throughput capacity of installations using other technologies for the OFMSW stabilization (including methane fermentation) did not exceed 5% [14]. 

The MBT technology still plays a major part in the management of the MSW in other EU countries, although the pace of building such installations is evidently winding down (National Waste Management Plan 2022). Around 570 MBT installations with a processing capacity of 55 million MG were operating in Europe at the end of 2016. Another 120 plants with a throughput capacity of 10 million tonnes per year will be put into service by 2025 [15].

Jędrczak and den Boer [16,17] carried out research on waste processed at 20 MBT installations in Poland, using diverse technologies for biostabilization of the OFMSW. They concluded that OFMSW accounted for 21.9% to 68.9% (48.5% on average) of the MSW mass processed in installations and contained glass in the amount of 4.1% to 26.3%, 10.9% on average. Glass (wet mass) in an amount between 16.2% and 87.3% went on to OFMSW. This means that in 2017, MBT plants in Poland landfilled nearly 400,000 tons of glass in stabilizer. Most of the glass contained in the stabilizer was packaging glass.

Cullet should, in the first place, be collected selectively and should not go to the MSW directed to an MBT installation or incineration plant. Considering the fact that MBT installations in Poland remain and will continue to be essential to ensure the reduction of landfilled biodegradable waste for years to come, the industrial-scale research has been carried out to recover recyclable glass from stabilizers sent to landfills. 

The pilot installation was built at the MBT plant in Marszów. Tests were carried out on stabilizer samples produced at the plant and collected from several other MBT plants in Poland. The goal of this paper was to assess the technical feasibility of recovery of glass contained in the stabilizer on a process line using processes commonly applied by the glass recycling industry.

## 2. Materials and Methods

The study was carried out in the period between March 12, 2019 and September 30, 2019. A total of 19 research sample series were used. In 13 series, the stabilizer produced in the installation was processed at the MBT plant in Marszów, and in six series, the stabilizer supplied by other plants located in different regions of Poland (MBT1–MBT 6: Gdańsk, Kryniczna, Ścinawka, Dąbrowa Górnicza, Stargard and Piotrowo) was processed.

The MBT plant in Marszów processes waste from 22 municipalities, inhabited by over 200,000 residents. In the mechanical part of the MBT installation, after the removal of waste requiring manual handling and after passing through a bag tearer, the waste is split into three fractions in a drum sieve with a mesh size of 80/280 mm. Fractions >280 mm and 80–280 mm are processed to produce fuel from waste. Subscreen fraction 0–80 mm is directed by the conveyor system to the aerobic biostabilization segment. The biological processing of waste is carried out in two stages. Stage One – intensive stabilization of waste in reinforced concrete bioreactors, with full automatic control of its course, duration: 3 weeks. Stage Two – maturing of waste in piles laid out in the open air for a period of 10–12 weeks, coupled with waste transfer [14]. Following the intensive stabilization, the waste was sent for research. In the final week was increased aeration for intensive drying of waste. 

Fraction <80 mm subjected to the intensive aerobic stabilization was also part of waste delivered from other MBT plants. Collection areas, the equipment used and the biological stabilization process parameters varied among these plants, which resulted in disparate characteristics of the tested samples.

The performance of the process line for cullet recovery was assessed in terms of the content and recovery level of materials (η) in the glass product (glass fraction). The material recovery level was calculated as a quotient of the material quantity in the product in relation to its content in the feed (stabilizer).

The characteristics of the tested waste samples are presented in Table 1.

The mass of the processed samples varied from 10.3 MG to 91.0 MG and amounted to 38.4 ± 19.5 MG on average. Samples taken from the Marszów MBT installation were very well dried out. Their moisture content was within the range of 6.0% to 28.0% (12.7 ± 6.6% on average). The moisture content of samples collected from other plants was from 9.6% to 32.0% (20.9 ± 9.6% on average). Stabilizers from the Marszów Waste Management Company contained glass in the amount of 17.3 ± 2.0% on average. The average share of glass in waste from other plants was 1.5 times lower.

## 3. Process Line and Equipment

### 3.1. The Technological Diagram of the Glass Recovery Line

**Figure 2:**
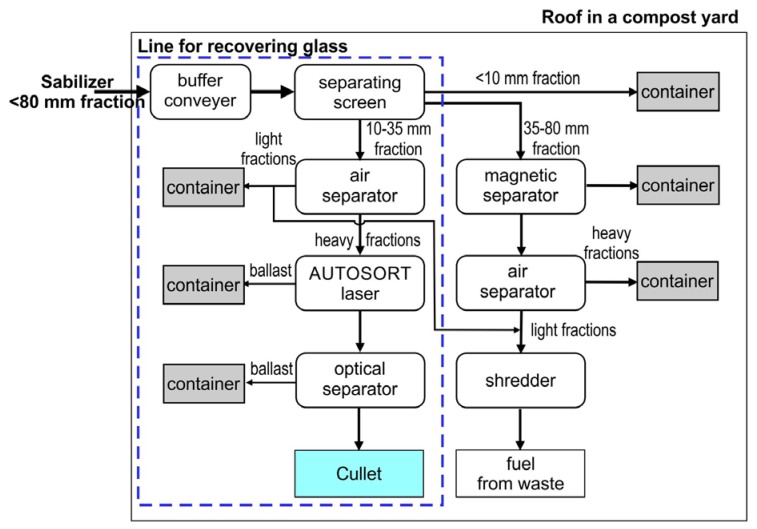
The technological diagram of the stabilizer processing line.

The process line for recovering glass from the stabilizer (biostabilized fraction 0–80 mm from municipal waste) was located under a roof in a compost yard (Figure 2). The line is an innovative solution. The authors are not aware of similar lines being described in literature sources. There are no such lines in over 192 existing installations in Poland.

**Figure 3:**
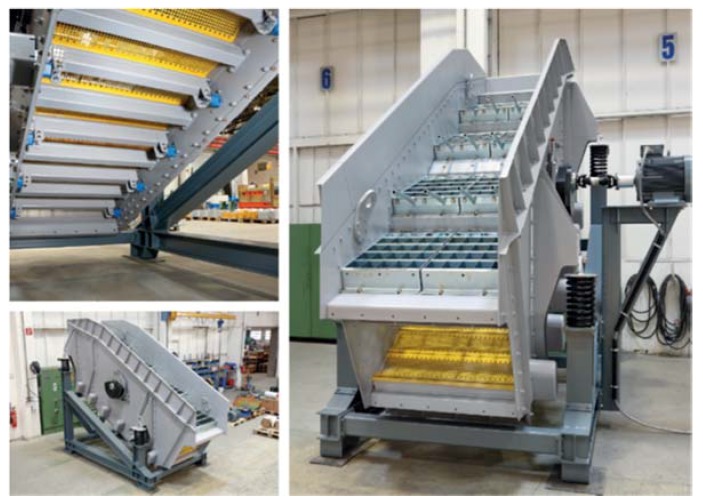
VARIOMAT double-deck screen (manufactured by IFE).

Once the biostabilization process is completed, the stabilizer is directed to a buffer conveyor with a capacity of 10 m^3^, with the use of a wheel loader. The waste is then fed by a conveyor belt from the buffer conveyor to a separating Flip-Flop screen with a flexible screening deck (Figure 3) manufactured by the company IFE (Waidhofen an der Ybbs, Austria). The screen separates the stabilizer into three fractions: 0–10 mm, 10–35 mm and 35–80 mm. The 0–10 mm fraction is transported by means of conveyor belts to a designated container.

**Figure 4:**
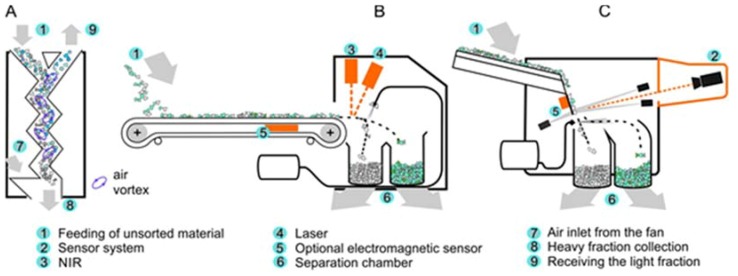
The equipment used in a process developed to recover glass from inert waste fractions using MBT. (**A**) ZIG ZAG air separator, (**B**) TOMRA AUTOSORT LASER separator, (**C**) TOMRA COMBISENSE CHUTE optical separator.

Fraction 10–35 mm is conveyed onto the ZIG ZAG air separator manufactured by company TRENSO TECHNIK (Weißenhorn, Germany, Figure 4A). In the air separator, fraction 10–35 mm is separated into light and heavy fractions. The light fraction is conveyed into a designated container, whereas the heavy fraction is transferred onto an accelerator belt, where it is transported to an AUTOSORT LASER device manufactured by the company TOMRA (Asker, Norway, Figure 4B). The AUTOSORT LASER device, which combines NIR and advanced laser technologies, enables the detection of glass and its separation from other waste. The separated glass fraction is transported by conveyor belts to a COMBISENSE CHUTE optical separator (TOMRA, Figure 4C), where other waste is further separated from glass. After removing metals by a magnetic separator, fraction 35–80 mm is split into light and heavy fractions in a NIHOT air separator (NIHOT, Amsterdam, Nederland). The heavy fraction is fed to a designated container, while the light fraction is fed directly to a LINDNER (LINDNER, Feistritz/Drau, Austria) shredder.

### 3.2. Devices

The VARIOMAT double-deck screen (manufactured by IFE), with the upper deck equipped with louver-type panels in a cascade arrangement, and protective divergating bars, a 35 mm cut point and the flexible Flip-Flop lower deck made of polyurethane mats with a 10 mm cut point, enables screening of materials that are very difficult to sieve and have a relatively high moisture content ranging from 15% to 20% (Figure 3). Each of the cascading panels of the screen deck is finished with a row of finger-like, divergating rods allowing large, flat waste to slide off them so that they do not clog the mesh in the sieve deck. The flexible Flip-Flop lower deck ensures effective (not less than 95%) sifting of fine, inert fractions such as: ash, earth and sand (after passing through the upper cascade deck).

On the bottom flexible deck, the screening machine ensures generation of 50 G force (50 × earth acceleration) for tossing waste, which guarantees high efficiency of sifting for the set fractions of stabilizer 0–80 mm and guarantees a self-cleaning effect of the sieve decks.

A ZIG ZAG air separator (Figure 4A) manufactured by the company TRENSO TECHNIK enables the separation of bulk materials of different specific weight of fraction 10–35 mm. The screened material is fed into the zigzag-shaped dispersal channel and distributed over its entire surface. The air used in the sorting process is generated by a fan and flows through the separator from its lower part upwards. The air current carries the lighter parts upwards, separating them from the input material, while the heavy fraction falls through the air stream.

The TOMRA AUTOSORT LASER separator (Figure 4B) separates the heavy fraction into two streams: waste rich in glass and ballast (impurities). The heavy fraction (10–35 mm) is evenly distributed on the sorter belt, where it is recognized by near infrared (NIR), laser and electromagnetic sensors. This separator allows for the separation of ceramics, stones, porcelain, metals and polymers, including transparent ones. If the sensors detect the material for sorting, they send a signal to the control unit to release the corresponding valves in the valve strip located at the end of the conveyor. The detected materials are separated from the rest by compressed air streams. 

The TOMRA COMBISENSE CHUTE optical separator (Figure 4C), is equipped with a high-resolution scanning camera with colour detection and an electromagnetic (EM) sensor. Thanks to the colour-detecting scanning camera, the material can be sorted according to colour, brightness, shape and size. The EM sensor sorts the material according to its electromagnetic conductivity. The feed material is evenly distributed onto the vibrating feeder and into the chute, where it is detected by the scanning camera. If the sensor detects the material to be sorted, it sends a signal to the control unit to release the corresponding valves in the valve strip at the end of the chute. The detected materials are separated from the rest by compressed air streams. The sorted material is divided into two fractions in the separation chamber: glass concentrate and ballast (impurities).

## 4. Results and Discussion

The results are shown and discussed for the intermediate product after processing in each machine to show the effect of each step of the process.

The mass of intermediate products obtained during the processing of stabilizers from Marszów and those collected from other MBT installations on the process line for the production of glass concentrate and their material composition are presented in Table 2.

**Figure 5:**
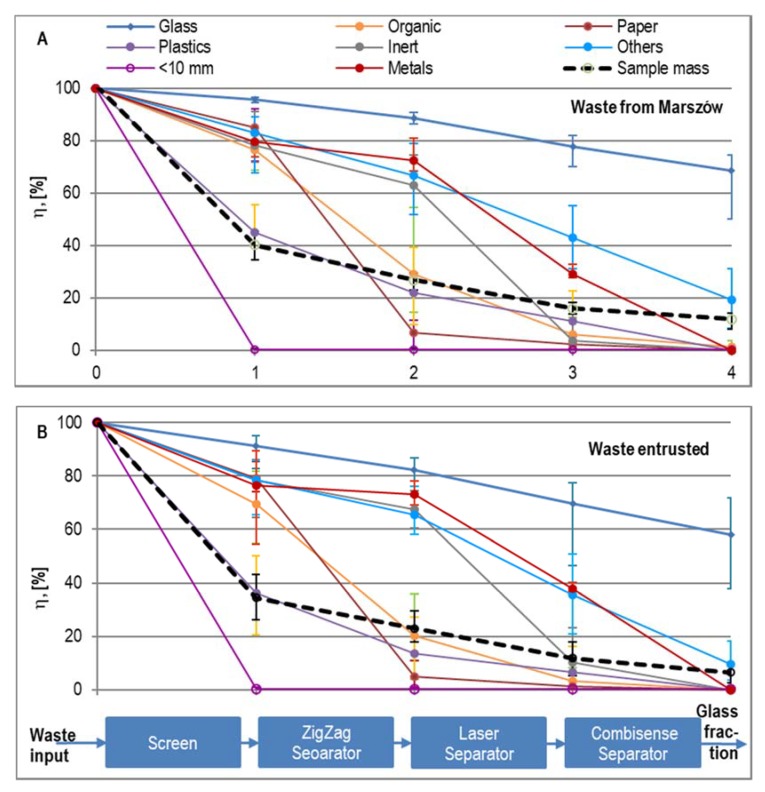
Recovery (η) of material components in the subsequent stages of the process of separating glass fractions from the stabilizer (average values: 13 series of waste from Marszów, 6 series of waste from other plants). (**A**) waste from Marszów (**B**) waste entrusted.

Figure 5 shows the recovery levels of material components from stabilizers in the subsequent stages of the glass concentrate production process (average values: waste from Marszów—13 series, waste from other plants—6 series). The VARIOMAT double-deck screen separates a stabilizer stream into three fractions: <10 mm, 10–35 mm and >35 mm. Fraction 10–35 mm directed to the production of glass concentrate constituted 39.5 ± 10.1% of the mass of stabilizers from Marszów and 33.6 ± 4.3% of the mass of stabilizers collected from other installations. Fraction <10 mm constituted respectively 50.2 ± 4.9% and 52.5 ± 13.3% of the stabilizers’ mass. It was a mixture of ash and sand, and also, in significant quantities, an organic fraction, which was dried and crushed to a large extent to grain <10 mm during the chambers unloading and transport of stabilizers to the screen. Significant losses on ignition (30.1 ± 5.0% DM on average) attest to the large amount of the organic fraction present.

The share of fraction >35 mm in the stabilizers’ mass was small–on average 8.9 ± 1.2% for the samples from Marszów and 13.8 ± 4.2% in the case of the other samples. This fraction mainly included plastics, with a 44.3 ± 5.9% and 63.7 ± 11.2% loss respectively, inerts (stones, rubble), with 21.8 ± 4.3% and 21.4 ± 3.7% losses respectively, and in the case of waste collected from other installations, 30.6 ± 2.3% loss for the organic fraction and 23.4 ± 1.7% for metals. 

The average loss of stabilizer mass in the screening process amounted to 66.5 ± 3.7% (samples from Marszów) and 65.5 ± 6.7% (other samples), but the loss of glass was relatively small, at 5.2 ± 0.7% and 8.7 ± 4.3% respectively.

The second unit in the line, the air separator, is quite effective in removing paper, organic matter and plastics from the remaining fraction stream with a grain size of 10–35 mm. The paper losses stood at 78.2 ± 5.1% and 74.1 ± 14.9% of their mass in the feed from the Marszów and other samples respectively, organic matter losses amounted to 54.3 ± 9.4% and 49.2 ± 19.7%, and plastics losses equalled 33.7 ± 5.1% and 22.8 ± 12.2%. The loss of glass was minor: 6.2 ± 1.3% and 9.0 ± 2.0% respectively for Marszow and other installations (Figure 5). The total loss of sample mass on the air separator was on average 7.7 ± 3.2% (samples from Marszów) and 11.4 ± 4.1% (other samples). 

Heavy fraction cleaning on the LASER separator was quite efficient in removing stones and rubble (59.3 ± 6.8% and 57.2 ± 5.6% loss of inerts), metals (43.2 ± 12.1% and 35.0 ± 15.9%) and "other components" (23.2 ± 9.0% and 29.9 ± 9.3%) in relation to their content in the feed. A 11.0 ± 3.3% loss of glass also occurred during the treatment of waste from Marszów and a 12.6 ± 7.7% loss in the case of waste from other plants. The total loss of sample mass in this separator amounted to, on average, 11.0 ± 3.3% during the treatment of waste from Marszów and 12.6 ± 7.7% in the case of waste from other plants. The total loss of sample mass on the air separator was on average 11.0 ± 2.1% (samples from Marszów) and 11.3 ± 6.5% (other samples).

Fraction 10–35 mm still remained heavily contaminated after passing through the air and LASER separators. Glass accounted for 83.6 ± 6.6% of the fraction mass in the case of Marszów stabilizers and only 71.2 ± 10.9% in the case of stabilizers from other MBT plants. The fraction contained a large percentage of unwanted components in the cullet, i.e.,: metals—3.7 ± 2.2% and 11.4 ± 5.9% respectively, inerts (stones, rubble, ceramics)—2.2 ± 1.9% and 7.6 ± 5.0%, organic components (organic matter, paper and plastics together)—6.9 ± 2.1% and 5.2 ± 2.1%, and “other components”—2.8 ± 1.2% and 4.1 ± 2.6%, respectively (Table 2).

Most of these components were removed in the COMBISENSE optical separator, the last device in the process line. The loss of metals comprised 29.2 ± 14.4% and 38.0 ± 22.9%, while the loss of "other components" was 24.4 ± 10.0% and 25.7±14.4%% respectively. The loss of glass was 8.9 ± 5.9% (samples from Marszów) and 11.6 ± 11.1%% (other samples). The total loss of sample mass in this separator was 4.0 ± 1.7% and 5.3 ± 3.0% on average in relation to the feed mass.

**Figure 6:**
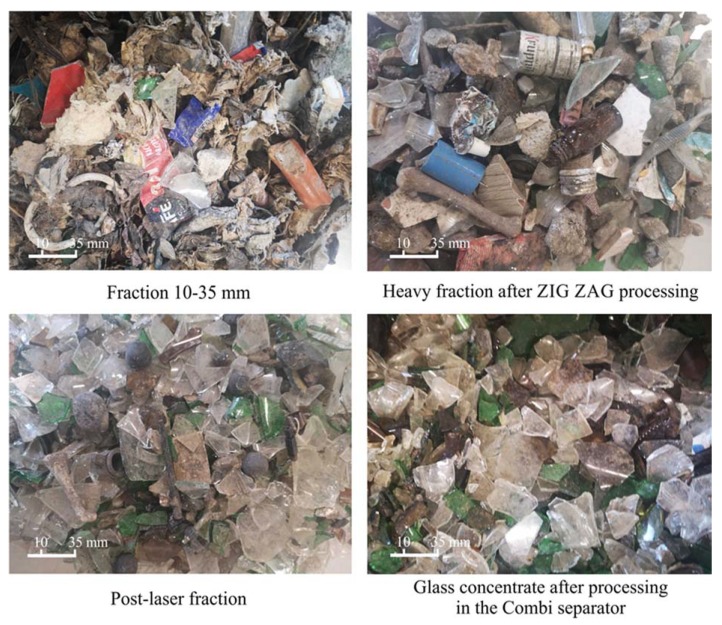
Fractions after successive stages of the process of recovering glass from stabilizers.

Figure 6 shows photos of the stabilizer and glass fractions after successive stages of the glass recovery process.

**Figure 7:**
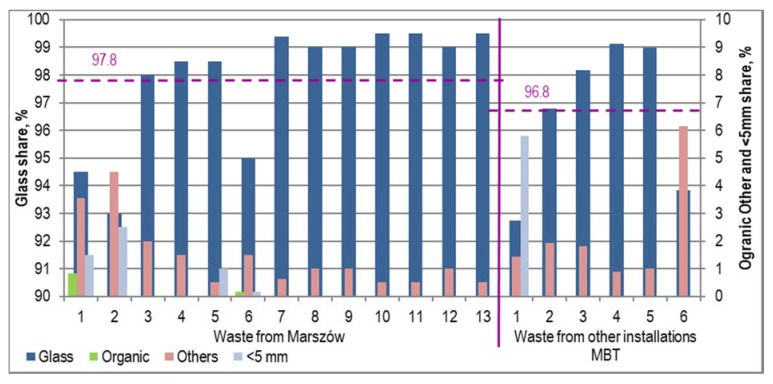
Composition of glass concentrates produced on the process line.

Processing the stabilizer on the line yielded between 49.8% and 74.3% of the glass contained in it (68.4 ± 7.0% on average) for the samples from Marszów and between 38.3% and 72.1% (58.4 ± 14.2% on average) in the case of samples collected from other MBT installations. Concentrates contained no paper, plastics or metals. Organic matter was found in only two samples from Marszów (Figure 7). Fraction <5 mm was found in 4 samples from Marszów, in the amount of from 0.2% to 2.5% and in one sample from other MBT installations (5.8%). “Other components” constituted the key contamination of the glass concentrate in all samples. The share of these components was on average 1.5 ± 1.3% in samples from Marszów and 2.2 ± 2,0% in the other samples. The glass content in the final product ranged from 93.0% to 99.5% (97.8 ± 2.2% on average) of the fraction mass in the case of stabilizers from Marszów and from 92.8% to 99.1% (96.8 ± 2.8% on average) in the case of stabilizers from other MBT installations (Figure 7). The share of glass in nine samples was 98% of sample weight. Glass contents lower than 98.0% were found in only five samples. These were samples obtained from stabilizers with a high moisture content. 

**Figure 8:**
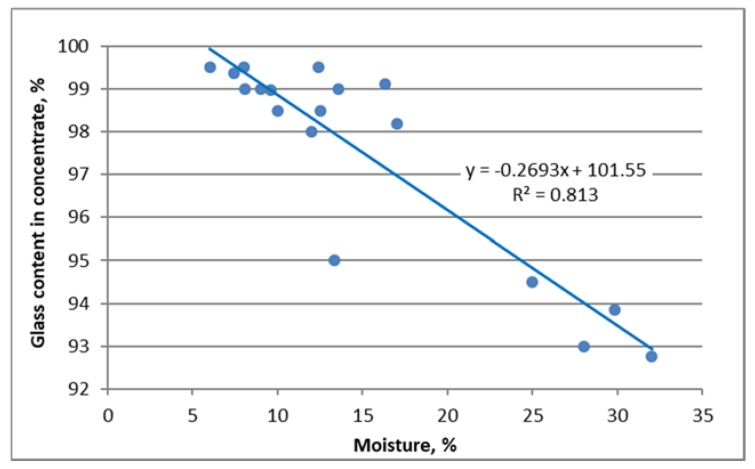
The dependence of the concentrate’s glass content on the stabilizer’s moisture content.

A linear relationship between the glass content in concentrate and moisture content was found (Figure 8). The correlation index (R2) for this relation was high and amounted to 0.813. Studies have found that as the moisture decreases (<15%), the possibility of cullet recovery increases to a value above 98%.

## 5. Conclusions

The stabilizer produced at MBT installations in Poland contains large quantities of packaging glass. This waste is currently mostly sent to landfills. A scheme of installations for recovering glass contained in stabilizer produced in MBT installations has been developed. The process included biodrying, screening, separation of light contaminants, separation of glass from rubble and stones, and optical sorting. A pilot plant was built in Marszów, where 19 different stabilizer samples were processed, including six samples collected from other MBT installations in Poland.

Processing the stabilizer on the designed line yielded between 49.8% and 74.3% of the glass contained in it (68.4 ± 7.0% on average) for the samples from Marszów and between 28.3% and 72.1% (58.4 ± 14.2% on average) for the samples obtained from other MBT installations. For most stabilizer samples (14 out of 19), excluding samples with a high moisture content, concentrate with glass content from 98.0% to 99.5% could be obtained. This product was accepted by glass recycling plants due to its low level of contamination with other materials and its appropriate particle size. It was shown that the quality of the concentrate largely depends on the stabilizer’s moisture content. The quality of the glass concentrate is determined by the process of stabilizer biodrying.

## Figures and Tables

**Table 1:** Types of plants and characteristic of tested waste.

Lp.	Plant MBT	Type of Building	Dates of Testing mm-dd		Sample Mass	Moisture	Morphological Composition							
							Glass	Organic	Paper	Plastics	Inert	Others	<10 mm	Metals
					[MG]	[%]	The Share of Wet Weight [%]							
1	MBT Marszów	mixed	03-12		91.0	25.0	14.8	3.6	7.0	7.0	6.8	1.1	58.4	1.4
2			03-25		58.6	28.0	15.5	2.9	8.5	7.2	10.3	1.3	53.0	1.3
3			04-08		53.8	12.0	16.1	3.7	8.8	8.5	7.7	1.2	51.5	2.6
4			04-24		53.7	12.5	19.1	3.1	7.2	8.0	9.6	0.7	51.4	1.0
5			05-06		50.5	10.0	14.6	2.4	6.9	5.2	10.5	0.8	57.3	2.3
6			05-23		48.0	13.3	15.3	2.6	8.7	6.2	8.1	1.1	55.5	2.5
7			06-04		37.2	7.4	20.1	3.1	8.8	6.1	9.1	0.9	49.9	2.1
8			06-18		45.5	13.6	17.8	3.2	10.1	7.8	12.5	1.0	45.2	2.4
9			07-03		34.8	9.0	16.3	3.3	8.6	8.2	11.0	1.0	49.6	2.0
10			07-17		46.0	8.0	18.4	4.0	12.7	8.4	8.7	0.9	45.3	1.7
11			07-30		36.8	12.4	20.0	3.8	11.8	8.3	8.1	1.0	44.7	2.2
12			08-13		40.1	8.1	17.3	3.6	9.6	8.0	10.2	1.1	47.8	2.5
13			08-27		29.2	6.0	19.3	4.1	10.9	9.1	9.9	1.2	43.2	2.2
I	Average values-samples of Marszów				48.1	12.7	17.3	3.3	9.2	7.5	9.4	1.0	50.2	2.0
	Standard deviation				15.5	6.6	2.0	0.5	1.8	1.1	1.6	0.2	4.9	0.5
	Minimum value				29.2	6.0	14.6	2.4	6.9	5.2	6.8	0.7	43.2	1.0
	Maximum value				91.0	28.0	20.1	4.1	12.7	9.1	12.5	1.3	58.4	2.6
14	MBT1	multi-family urban		04-10	19.3	32.0	12.1	3.7	9.9	8.9	11.0	1.7	49.6	3.1
15	MBT2	rural		05-21	10.3	-	14.6	2.8	11.2	5.6	10.6	1.0	49.5	4.6
16	MBT3	mixed		05-21	12.0	17.0	12.9	1.8	6.3	4.7	10.3	1.2	57.7	4.9
17	MBT4	mixed		05-22	21.0	16.3	9.6	2.1	5.8	5.1	7.2	0.6	67.4	2.1
18	MBT5	mixed		09-09	20.2	9.6	11.2	2.3	6.7	6.4	9.1	0.7	61.5	2.1
19	MBT6	mixed		09-30	22.0	29.8	7.8	5.5	10.3	16.9	23.4	1.9	29.2	4.8
II	Average values-samples entrusted				17.5	20.9	11.4	3.1	8.4	7.9	11.9	1.2	52.5	3.6
	Standard deviation				5.0	9.6	2.4	1.4	2.4	4.7	5.8	0.5	13.3	1.3
	Minimum value				10.3	9.6	7.8	1.8	5.8	4.7	7.2	0.6	29.2	2.1
	Maximum value				22.0	32.0	14.6	5.5	11.2	16.9	23.4	1.9	67.4	4.9

**Table 2:** Mass and material composition of intermediate products obtained in the subsequent stages of the process of treating the stabilizer from Marszów and that collected from other MBT installations on the glass concentrate production line.

Waste Stream	Fraction Mass [MG]	Share of Component, [%]							
		Glass	Organic	Paper	Plastics	Inert	Others	<10 mm	Metals
**Waste from Marszow**									
Stabilizer	48.1 ± 15.5	17.3 ± 2.0	3.3 ± 0.5	9.2 ± 1.8	7.5 ± 1.1	9.4 ± 1.6	1.0 ± 0.2	50.2±4.9	2.0 ± 0.5
Fraction 10–35 mm	19.0 ± 4.7	41.0 ± 2.9	6.3 ± 1.0	19.3 ± 2.6	8.4 ± 1.6	18.4 ± 3.5	2.2 ± 0.5	0.4 ± 0.3	4.0 ± 1.2
Heavy fraction after ZIG ZAG	12.8 ± 3.8	57.0 ± 5.2	3.6 ± 1.6	2.2 ± 0.9	6.1 ± 2.6	22.3 ± 5.0	2.7 ± 0.7	0.6 ± 0.5	5.5 ± 1.7
Laser fraction	7.6 ± 2.2	83.6 ± 6.6	1.0 ± 0.6	1.1 ± 0.7	4.8 ± 3.0	2.2 ± 1.9	2.8 ± 1.2	1.0 ± 0.7	3.7±2.2
Glass fraction	5.7 ± 1.8	97.9 ± 2.2	0.0	0.0	0.0	0.0	1.5 ± 1.3	0.7 ± 1.2	0.0
**Entrusted Waste (MBT1 – MBT6)**									
Stabilizer	17.5 ± 5.0	11.4 ± 2.4	3.1 ± 1.4	8.4 ± 2.4	7.9 ± 4.7	11.9 ± 5.8	1.2 ± 0.5	52.5 ± 13.3	3.6 ± 1.3
Fraction 10–35 mm	5.9 ± 1.7	30.9 ± 7.4	5.9 ± 1.4	18.9 ±3.3	7.4 ± 1.7	26.2 ± 6.7	2.7 ± 0.8	0.2 ± 0.5	7.9 ± 2.3
Heavy fraction after ZIG ZAG	4.0 ± 1.4	42.4 ± 12.0	2.9 ± 2.4	1.7 ± 1.3	4.5 ± 2.9	33.4 ± 7.4	3.4 ± 1.2	0.3 ± 0.8	11.3 ± 3.6
Laser fraction	1.9 ± 0.4	71.2 ± 10.9	0.8 ± 0.6	0.8 ± 0.7	3.6 ± 2.9	7.6 ± 5.0	4.1 ± 2.6	0.6 ± 1.5	11.4 ± 5.9
Glass fraction	1.1 ± 0.3	96.8 ± 2.8	0.0	0.0	0.0	0.0	2.2 ± 2.0	1.0 ± 2.4	0.0
